# Modelling the Effect of SPION Size in a Stent Assisted Magnetic Drug Targeting System with Interparticle Interactions

**DOI:** 10.1155/2015/618658

**Published:** 2015-03-01

**Authors:** Adil Mardinoglu, P. J. Cregg

**Affiliations:** ^1^Department of Biology and Biological Engineering, Chalmers University of Technology, 412 96 Gothenburg, Sweden; ^2^Materials Characterisation & Processing Group, Waterford Institute of Technology, Waterford, Ireland

## Abstract

Cancer is a leading cause of death worldwide and it is caused by the interaction of genomic, environmental, and lifestyle factors. Although chemotherapy is one way of treating cancers, it also damages healthy cells and may cause severe side effects. Therefore, it is beneficial in drug delivery in the human body to increase the proportion of the drugs at the target site while limiting its exposure at the rest of body through Magnetic Drug Targeting (MDT). Superparamagnetic iron oxide nanoparticles (SPIONs) are derived from polyol methods and coated with oleic acid and can be used as magnetic drug carrier particles (MDCPs) in an MDT system. Here, we develop a mathematical model for studying the interactions between the MDCPs enriched with three different diameters of SPIONs (6.6, 11.6, and 17.8 nm) in the MDT system with an implanted magnetizable stent using different magnetic field strengths and blood velocities. Our computational analysis allows for the optimal design of the SPIONs enriched MDCPs to be used in clinical applications.

## 1. Introduction

Cancer is a leading cause of death worldwide. Its cause is multifactorial and is linked to the interaction of genomic, environmental, and lifestyle factors [1]. Cancer patients are often diagnosed with localized reduction or loss of cellular control and normal maturation mechanisms that incorporate excessive cell growth, loss of cell differentiation, and the ability of cancerous tissue to grow into neighbouring tissues [2, 3]. Chemotherapy is one type of cancer treatment that inhibits the growth of, or kills, tumours. However, chemotherapy can damage healthy cells in the human body and it has many undesirable side effects [4]. It is therefore beneficial to alter the distribution of drugs in the human body, increasing the proportion of drugs at the target site while limiting concentration and effects in the rest of body through the use of Magnetic Drug Targeting (MDT) [5, 6]. Similar techniques have also been used to deliver other agents including cells [7].

MDT refers to the attachment of therapeutics to magnetizable particles to concentrate them at the desired locations by applying magnetic fields [8]. It includes the investigation of an external magnetic field and its interaction with biocompatible magnetic drug carrier particles (MDCPs) [9]. Significant difficulties in MDT are the inherently weak magnetic force relative to the hydrodynamic forces and targeting zones deep below the skin [10, 11]. This makes MDCP collection problematic, because the magnetic force on a MDCP is proportional not only to the magnitude of the magnetic field but also to its gradient. To overcome these limitations, soft ferromagnetic materials such as wires, seeds, and stents are implanted into the body to increase the localized magnetic field strength and gradient, and this technique is called Implant Assisted Magnetic Drug Targeting (IA-MDT) [12–15]. Different theoretical and clinical applications of IA-MDT have been developed [16–23]. Moreover, an IA-MDT system which uses a magnetizable stent as an implant and high gradient magnetic separation (HGMS) in a physiologically stretched vessel was studied with a 2D mathematical model [24]. In this Stent Assisted Magnetic Drug Targeting (SA-MDT) system, a ferromagnetic stent was implanted to aid collection of MDCPs in an elastic tube that has similar mechanical properties to the blood vessel and the changes in the mechanical behaviour were analyzed under the influence of mechanical forces generated.

There has been a growing interest in the scientific and clinical application of MDCPs as MDT vehicles for the development of efficient treatment strategies. A nanoparticle-based cancer drug has been developed and the phase 1 clinical study of cancer patients providing positive clinical evidence for the progress of nanoparticle application is reported [25]. Superparamagnetic iron oxide nanoparticles (SPIONs) have also been used as magnetic resonance imaging (MRI) contrast agents for labelling mammalian cells since their features can be easily tailored to include targeting moieties, fluorescence dyes, or therapeutic agents [26]. SPIONs can also be taken up by the cells through endocytosis and one particular SPION that contains ferumoxides is approved for hepatic imaging by the US Food and Drug Administration (FDA) [27].

The fraction of the constituting atoms on the surface of the nanoparticles varies with the decrease in the size of the particles (<100 nm) and this leads to significant changes in the magnetic structure and properties of the nanophase materials. The variation of the magnetic behaviour of well-dispersed monodisperse Fe_3_O_4_ nanoparticles with respect to particle diameters (6.6, 11.6, and 17.8 nm) has been previously investigated [28]. It has been reported that the largest particles are ferromagnetic at room temperature and smaller nanoparticles exhibit superparamagnetism with the blocking temperatures increasing with the particle size. Saturation magnetization of the oleate-capped Fe_3_O_4_ nanoparticles with different diameters at 300 K is shown in Table 1.

**Figure 1:**
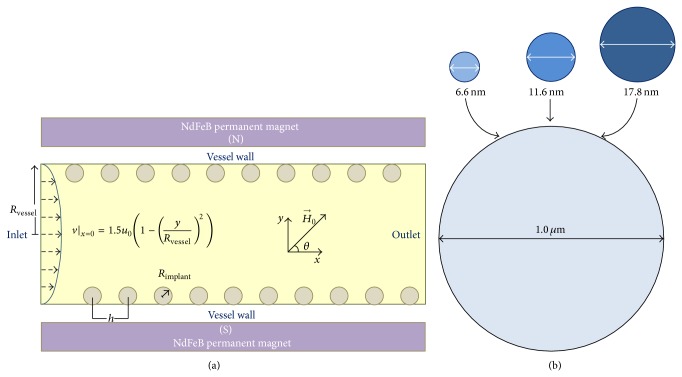
(a) Schematic of the CV used for studying the behaviour of SPIONs enriched MDCPs in SA-MDT system. (b) The modified magnetic force acting on MDCPs enriched with three different sizes of SPIONs is modelled in the SA-MDT system using different magnetic field strengths and blood velocities.

Here, we propose SPIONs as carriers in SA-MDT system using a magnetizable stent as an implant and focus on the theoretical modelling of the interaction between the MDCPs enriched with three different sizes of SPIONs (see Figure 1). The quantity of the SPIONs included in the MDCPs is inversely proportional to the diameter in SPIONs in each simulation. The quantity of the carrier particles at the desired site under the influence of four different magnetic field strengths and four different blood velocities is considered.

## 2. Defining the SA-MDT System

In order to optimize the size of the SPIONs in the content of MDCPs in an SA-MDT system, a 2D mathematical model was developed based on a previous model [18]. The mathematical model geometry of the SA-MDT system comprises a magnetizable coiled wire stent implant in close contact with the inner wall of a biological vessel. We expand the previous model [18] and present the next experimental challenge to develop a clinically relevant SA-MDT system. We model the behaviour of *N* MDCPs under the influence of (i) Stokes drag, (ii) hydrodynamic interaction forces, and (iii) magnetic forces that account for the mutual magnetic dipole-dipole interactions and calculated (iv) the velocity of each MDCP and MDCP_*n*_ and (v) the system performance in terms of collection efficiency (CE) ignoring the effect of inertia and gravity (Figure 1).

The forces acting on a given particle labeled  *n* MDCP_*n*_ are calculated as follows.

(i) Stokes drag, ${\vec{F}}_{s_{n}}$, decreases the blood velocity relative to the MDCPs and it can be written as
  (1) $$\begin{array}{c} {\vec{F}}_{s_{n}}=6\pi \eta_{\text{blood}}R_{p_{n}}\left({\vec{v}}_{\text{blood}}-{\vec{v}}_{p_{n}}\right), \end{array}$$
  where *η*_blood_ is the viscosity of the blood, *R*_*p*_*n*__ is the radius of MDCP_*n*_, and ${\vec{v}}_{\text{blood}}$ and ${\vec{v}}_{p_{n}}$ are the velocities of the blood and MDCP_*n*_, respectively. In the model, ${\vec{v}}_{\text{blood}}$ is determined by solving the appropriate Navier–Stokes equations as previously described [15].
(ii) Hydrodynamic interaction force, ${\vec{F}}_{\text{hy}{\text{d}}_{n}}$, is determined as the disturbance of the MDCP_*n*_ due to the movement of other MDCPs in the blood flow. By considering *N* MDCPs, the force acting on MDCP_*n*_ due to the presence of the other (*N* − 1) MDCPs is given by
  (2) $$\begin{array}{c} {\vec{F}}_{\text{hy}{\text{d}}_{n}}=\overset{N}{\underset{\left(\begin{array}{c} i=1\\ i\neq n \end{array}\right)}{\sum }}\xi_{ni}\cdot \left({\vec{v}}_{\text{blood}}-{\vec{v}}_{p_{i}}\right), \end{array}$$
 where ${\vec{v}}_{p_{i}}$ is the velocity of MDCP_*i*_ and *ξ*_*ni*_ is the modification due to the hydrodynamic interaction.
(iii) Magnetic forces acting on MDCP_*n*_, ${\vec{F}}_{mm_{n}}$, account for the externally applied magnetic field strength and mutual magnetic dipole-dipole interactions between MDCPs. Considering the *N* MDCPs, each MDCP is taken as spherical and is having a homogeneous magnetic flux throughout the entire volume. ${\vec{F}}_{mm_{n}}$ on MDCP_*n*_ can be written as
  (3) $$\begin{array}{c} {\vec{F}}_{mm_{n}}=\left({\vec{m}}_{n}\cdot \nabla \right){{\vec{B}}_{\text{Total}}}_{n}, \end{array}$$
 where ${\vec{m}}_{n}$ is the total magnetic moment of MDCP_*n*_ and ${{\vec{B}}_{\text{Total}}}_{n}$ is the total magnetic flux acting on MDCP_*n*_.
(iv) The velocity of MDCP_*n*_, ${\vec{v}}_{p_{n}}$, is obtained by summing the Stokes drag, the force due to hydrodynamic interaction, and the modified magnetic force (ignoring the inertial forces) as
  (4) 6πηbloodRpnv→blood−v→pn+∑i=1i≠nNξni·v→blood−v→pi +m→n·∇B→Totaln=0.
(v) The system performance of the model is calculated in terms of collection efficiency (CE) and the trajectories of MDCP_*n*_ are obtained from evaluating the streamline functions. Consider
  (5) $$\begin{array}{c} \text{CE}=\frac{2R_{\text{vessel}}-y_{1}+y_{2}}{2R_{\text{vessel}}}100\%, \end{array}$$
 where *R*_vessel_ is the radius of the vessel and *y*_1_ and *y*_2_ are defined by the location of the streamline at the entrance of control volume (CV) of the last MDCPs captured by the stent wires.

## 3. Results

In the current study, we present the simulation results of the behaviour of MDCPs enriched with three different sizes of SPIONs (diameters 6.6, 11.6, and 17.8 nm) in SA-MDT system. We examine the effects of interactions on the CE of the system in terms of the changes in blood velocity and applied magnetic field strength (Table 2).

We calculate the forces due to the magnetic dipole-dipole and hydrodynamic interactions on *N* (*N* = 100) MDCPs together with the blood flow velocity. Magnetic and hydrodynamic forces acting on MDCPs as well as blood velocity were calculated using the finite volume library OpenFOAM [29]. We create a homogeneously distributed square cloud of 100 MDCPs at the entrance of the CV, place the centre of the cloud at boundary of the reference capture cross section, simulate the behaviour of MDCPs at every time step considering their agglomeration, and eventually obtain the altered trajectories of MDCPs for calculating the CE. The number of the MDCPs is limited to 100 and the effective initial distance between the MDCPs at the entrance of CV is presented in Table 2 as calculated from a previous experimental setup [18]. In our previous studies, the number of MDCPs in the simulations has been limited to 25 leading to close agreement with the experimental results [18].

**Figure 2:**
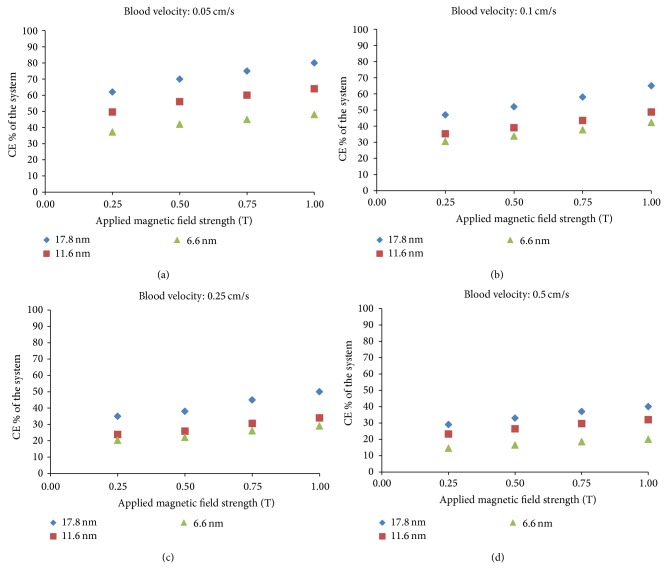
CE of the SA-MDT system is plotted with different magnetic field strengths (0.25, 0.50, 0.75, and 1 T) and blood velocities (0.05, 0.1, 0.25, and 0.5 cm/s).

In order to describe the effect of different SPION diameter on the content of MDCPs, ${\vec{F}}_{mm_{n}}$is calculated at the entrance of CV for each MDCP and presented in Figure 2. The saturation magnetization of SPIONs (oleate-capped, Fe_3_O_4_ nanoparticles) [28] is presented in Table 1. The number of the SPIONs in MDCP is inversely proportional to the diameter of the SPIONs. Improving the content and structure of the MDCPs and having better surface to volume ratio, we can have better applications of MDCPs in clinical studies.

Figure 2 shows the variation in CE of the SA-MDT system at four different applied magnetic field strengths (0.25, 0.50, 0.75, and 1 T) and four different injection fluid velocities (0.05, 0.1, 0.25, and 0.5 cm/s). The resulting collection efficiencies derived from this mathematical model are in agreement with previously published work [18], and with differing SPION diameter, the system performance can differ by up to 20% in absolute terms.

## 4. Discussions

We have presented SA-MTD model incorporating the agglomeration of particles known to occur in real biological systems and studied the effect of SPION diameter used in the MDCPs using different magnetic field strengths and blood velocities. We calculated both the dipole-dipole and hydrodynamic interactions for 100 particles and the resulting collection efficiencies derived from the mathematical model are in closer agreement with our latest experimental results [18].

We envisage that new insights obtained from the results of our analysis may be used in prediction of efficacy of targeted drug delivery for designing effective nanotherapeutic tools that can translate into the clinic. The CE of the system is increased with the higher magnetic field strength and decreased with the higher blood velocities as expected. Moreover, the modelling of different sizes of SPIONs in a SA-MDT system presented in this work represents a useful analytical tool for the prediction of the efficacy of targeted drug delivery. Our simulations indicate that size of the SPIONs in MDCPs together with saturation magnetization of the SPIONs has considerable effect on collection efficiency of the SA-MDT system. The response of SA-MDT is mainly dominated by the size of SPIONs and the saturation magnetization value of SPIONs, and these parameters can be calibrated based on the clinical applications of SA-MDT system using the results of our simulation. Improvement of the fundamental models in MDT systems may allow for the development of the more complex models that include systems level interactions.

The presented mathematical model for the movement of the MDCPs in the blood can be integrated with genome-scale metabolic models (GEMs) for healthy cells/tissues [30–33], cancers [34, 35], and cancer cell lines [36]. GEMs are the compilation of biochemical reactions to define the entire known metabolism of the cells and tissues [37, 38], and they are reconstructed through the integration of proteomics [33, 39], transcriptomics [40–42], and metabolomics data [43]. Such integrative models can be used for discovery of novel biomarkers as well as for identification of drug targets to develop efficient treatment strategies for metabolism related diseases including cancer [44].

## Figures and Tables

**Table 1:** Saturation magnetization (*M*_*s*_) of the oleate-capped Fe_3_O_4_ nanoparticles with different diameters at 300 K.

Diameter (nm)	Saturation magnetization (kA/m)
6.6	15.7
11.6	14.7
17.8	3.4

**Table 2:** Blood and material parameters used in the SA-MDT system.

Properties	Symbol	Units	Values
Applied field properties			
Magnitude	*μ*_0_ *H*_0_	T	0.25, 0.50, 0.75, 1
Angle of field direction	*θ*	—	*π*/2
Physical properties			
Temperature	*T*	K	300
Boltzmann's constant	*k*_*B*_	J/K	1.38 × 10^23^
Permeability of vacuum	*μ*_0_	Tm/A	4*π* × 10^−7^
MDCPs properties			
Polymer material	—	—	P(S/V-COOH)Mag
Radius	*R*_*p*_	*μ*m	0.5
MDCP concentration	—	Particle/L	4 × 10^10^
Density of the polymer material	*ρ*_pol,*p*_	kg/m^3^	950
Initial distance between MDCPs	—	*μ*m	29.24
Saturation magnetization	*M*_*p*,*s*_	kA/m	22.4
Stent properties			
Material	—	—	SS 430
Wire radius	*R*_wire_	*μ*m	62.5
Loop separation	*H*	cm	0.2
Number of loops	*N*_*l*_	—	10
Coil length	*L*	cm	2
Saturation magnetization	*M*_implant,*s*_	kA/m	1261
Magnetic susceptibility	*X*_implant,0_	—	1000
Blood and vessel properties			
Velocity	*u*_0_	cm/s	0.05, 0.1, 0.25, 0.5
Volume	*V*_blood_	mL	10
Density	*ρ*_*b*_	kg/m^3^	1000
Viscosity	*η*_*b*_	kg/ms	1.0 × 10^−3^
Vessel radius	*R*_vessel_	cm	0.05
Magnetic material properties			
Material	—	—	Oleate-capped Fe_3_O_4_
Weight content	*X*_fm,*p*_	wt%	100
Density	*ρ*_fm,*p*_	kg/m^3^	5000
Magnetic moment	*m*_fm,*p*_	Am^2^	
Saturation magnetization	*M*_fm,*p*,*s*_	kA/m	See Table 1
Diameter	*R*_fm,*p*_	nm	See Table 1

## References

[1] Hanahan D., Weinberg R. A. (2011). Hallmarks of cancer: the next generation. *Cell*.

[2] Vander Heiden M. G. (2011). Targeting cancer metabolism: a therapeutic window opens. *Nature Reviews Drug Discovery*.

[3] Tennant D. A., Durán R. V., Gottlieb E. (2010). Targeting metabolic transformation for cancer therapy. *Nature Reviews Cancer*.

[4] Kaufmann S. H., Earnshaw W. C. (2000). Induction of apoptosis by cancer chemotherapy. *Experimental Cell Research*.

[5] Kempe H., Kempe M. (2010). The use of magnetite nanoparticles for implant-assisted magnetic drug targeting in thrombolytic therapy. *Biomaterials*.

[6] Lübbe A. S., Alexiou C., Bergemann C. (2001). Clinical applications of magnetic drug targeting. *Journal of Surgical Research*.

[7] Riegler J., Wells J. A., Kyrtatos P. G., Price A. N., Pankhurst Q. A., Lythgoe M. F. (2010). Targeted magnetic delivery and tracking of cells using a magnetic resonance imaging system. *Biomaterials*.

[8] Shapiro B., Kulkarni S., Nacev A., Muro S., Stepanov P. Y., Weinberg I. N. (2014). Open challenges in magnetic drug targeting. *Wiley Interdisciplinary Reviews: Nanomedicine and Nanobiotechnology*.

[9] Lübbe A. S., Bergemann C., Riess H. (1996). Clinical experiences with magnetic drug targeting: a phase I study with 4'-epidoxorubicin in 14 patients with advanced solid tumors. *Cancer Research*.

[10] Iacob G., Rotariu O., Strachan N. J. C., Häfeli U. O. (2004). Magnetizable needles and wires—modeling an efficient way to target magnetic microspheres in vivo. *Biorheology*.

[11] Nacev A., Weinberg I. N., Stepanov P. Y. (2015). Dynamic inversion enables external magnets to concentrate ferromagnetic rods to a central target. *Nano Letters*.

[12] Yellen B. B., Forbes Z. G., Halverson D. S. (2005). Targeted drug delivery to magnetic implants for therapeutic applications. *Journal of Magnetism and Magnetic Materials*.

[13] Rosengart A. J., Kaminski M. D., Chen H. T., Caviness P. L., Ebner A. D., Ritter J. A. (2005). Magnetizable implants and functionalized magnetic carriers: a novel approach for noninvasive yet targeted drug delivery. *Journal of Magnetism and Magnetic Materials*.

[14] Ritter J. A., Ebner A. D., Daniel K. D., Stewart K. L. (2004). Application of high gradient magnetic separation principles to magnetic drug targeting. *Journal of Magnetism and Magnetic Materials*.

[15] Mardinoglu A. (2009). *Inclusion of interactions in mathematical modelling of implant assisted magnetic drug targeting [Ph.D. thesis]*.

[16] Cregg P. J., Murphy K., Mardinoglu A. (2008). Calculation of nanoparticle capture efficiency in magnetic drug targeting. *Journal of Magnetism and Magnetic Materials*.

[17] Cregg P. J., Murphy K., Mardinoglu A. (2009). Inclusion of magnetic dipole-dipole and hydrodynamic interactions in implant-assisted magnetic drug targeting. *Journal of Magnetism and Magnetic Materials*.

[18] Cregg P. J., Murphy K., Mardinoglu A., Prina-Mello A. (2010). Many particle magnetic dipole-dipole and hydrodynamic interactions in magnetizable stent assisted magnetic drug targeting. *Journal of Magnetism and Magnetic Materials*.

[19] Cregg P. J., Murphy K., Mardinoglu A. (2012). Inclusion of interactions in mathematical modelling of implant assisted magnetic drug targeting. *Applied Mathematical Modelling*.

[20] Chen H., Ebner A. D., Kaminski M. D., Rosengart A. J., Ritter J. A. (2005). Analysis of magnetic drug carrier particle capture by a magnetizable intravascular stent—2: parametric study with multi-wire two-dimensional model. *Journal of Magnetism and Magnetic Materials*.

[21] Avilés M. O., Ebner A. D., Ritter J. A. (2007). Ferromagnetic seeding for the magnetic targeting of drugs and radiation in capillary beds. *Journal of Magnetism and Magnetic Materials*.

[22] Avilés M. O., Ebner A. D., Ritter J. A. (2008). Implant assisted-magnetic drug targeting: comparison of in vitro experiments with theory. *Journal of Magnetism and Magnetic Materials*.

[23] Mangual J. O., Avilés M. O., Ebner A. D., Ritter J. A. (2011). *In vitro* study of magnetic nanoparticles as the implant for implant assisted magnetic drug targeting. *Journal of Magnetism and Magnetic Materials*.

[24] Mardinoglu A., Cregg P. J., Murphy K., Curtin M., Prina-Mello A. (2011). Theoretical modelling of physiologically stretched vessel in magnetisable stent assisted magnetic drug targeting application. *Journal of Magnetism and Magnetic Materials*.

[25] Sheridan C. (2012). Proof of concept for next-generation nanoparticle drugs in humans. *Nature Biotechnology*.

[26] Lee J.-H., Schneider B., Jordan E. K., Liu W., Frank J. A. (2008). Synthesis of complexable fluorescent superparamagnetic iron oxide nanoparticles (FL SPIONs) and cell labeling for clinical application. *Advanced Materials*.

[27] de Vries I. J. M., Lesterhuis W. J., Barentsz J. O. (2005). Magnetic resonance tracking of dendritic cells in melanoma patients for monitoring of cellular therapy. *Nature Biotechnology*.

[28] Caruntu D., Caruntu G., O'Connor C. J. (2007). Magnetic properties of variable-sized Fe_3_O_4_ nanoparticles synthesized from non-aqueous homogeneous solutions of polyols. *Journal of Physics D: Applied Physics*.

[29] Jasak H. (2009). OpenFOAM: open source CFD in research and industry. *International Journal of Naval Architecture and Ocean Engineering*.

[30] Mardinoglu A., Agren R., Kampf C. (2013). Integration of clinical data with a genome-scale metabolic model of the human Adipocyte. *Molecular Systems Biology*.

[31] Mardinoglu A., Agren R., Kampf C., Asplund A., Uhlen M., Nielsen J. (2014). Genome-scale metabolic modelling of hepatocytes reveals serine deficiency in patients with non-alcoholic fatty liver disease. *Nature Communications*.

[32] Mardinoglu A., Kampf C., Asplund A. (2014). Defining the human adipose tissue proteome to reveal metabolic alterations in obesity. *Journal of Proteome Research*.

[33] Uhlen M., Fagerberg L., Hallstrom B. M. (2015). Tissue-based map of the human proteome. *Science*.

[34] Agren R., Bordel S., Mardinoglu A., Pornputtapong N., Nookaew I., Nielsen J. (2012). Reconstruction of genome-scale active metabolic networks for 69 human cell types and 16 cancer types using INIT. *PLoS Computational Biology*.

[35] Agren R., Mardinoglu A., Asplund A., Kampf C., Uhlen M., Nielsen J. (2014). Identification of anticancer drugs for hepatocellular carcinoma through personalized genome-scale metabolic modeling. *Molecular Systems Biology*.

[36] Ghaffari P., Mardinoglu A., Asplund A. (2015). Identifying anti-growth factors for human cancer cell lines through genome-scale metabolic modeling. *Scientific Reports*.

[37] Mardinoglu A., Gatto F., Nielsen J. (2013). Genome-scale modeling of human metabolism—a systems biology approach. *Biotechnology Journal*.

[38] Mardinoglu A., Nielsen J. (2012). Systems medicine and metabolic modelling. *Journal of Internal Medicine*.

[39] Uhlen M., Oksvold P., Fagerberg L. (2010). Towards a knowledge-based Human Protein Atlas. *Nature Biotechnology*.

[40] Kampf C., Mardinoglu A., Fagerberg L. (2014). The human liver-specific proteome defined by transcriptomics and antibody-based profiling. *The FASEB Journal*.

[41] Kampf C., Mardinoglu A., Fagerberg L. (2014). Defining the human gallbladder proteome by transcriptomics and affinity proteomics. *Proteomics*.

[42] Fagerberg L., Hallstrom B. M., Oksvold P. (2014). Analysis of the human tissue-specific expression by genome-wide integration of transcriptomics and antibody-based proteomics. *Molecular and Cellular Proteomics*.

[43] Wishart D. S., Knox C., Guo A. C. (2009). HMDB: a knowledgebase for the human metabolome. *Nucleic Acids Research*.

[44] Mardinoglu A., Nielsen J. (2015). New paradigms for metabolic modeling of human cells. *Current Opinion in Biotechnology*.

